# Fast and quantitative mitophagy assessment by flow cytometry using the *mito*-QC reporter

**DOI:** 10.3389/fcell.2024.1460061

**Published:** 2024-09-11

**Authors:** Juan Ignacio Jiménez-Loygorri, Carlos Jiménez-García, Álvaro Viedma-Poyatos, Patricia Boya

**Affiliations:** ^1^ Department of Cellular and Molecular Biology, Centro de Investigaciones Biológicas Margarita Salas, CSIC, Madrid, Spain; ^2^ Department of Neuroscience and Movement Science, Faculty of Science and Medicine, University of Fribourg, Fribourg, Switzerland

**Keywords:** FACS, mitochondria, autophagy, retina, SI, Fisetin, phenanthroline

## Abstract

Mitochondrial quality control is finely tuned by mitophagy, the selective degradation of mitochondria through autophagy, and mitochondrial biogenesis. Removal of damaged mitochondria is essential to preserve cellular bioenergetics and prevent detrimental events such as sustained mitoROS production, pro-apoptotic cytochrome c release or mtDNA leakage. The array of tools available to study mitophagy is very limited but in constant development. Almost a decade ago, we developed a method to assess mitophagy flux using MitoTracker Deep Red in combination with lysosomal inhibitors. Now, using the novel tandem-fluorescence reporter *mito*-QC (mCherry-GFP-FIS1^101−152^) that allows to differentiate between healthy mitochondria (mCherry^+^GFP^+^) and mitolysosomes (mCherry^+^GFP^−^), we have developed a robust and quantitative method to assess mitophagy by flow cytometry. This approach has been validated in ARPE-19 cells using PINK1/Parkin-dependent (CCCP) and PINK1/Parkin-independent (DFP) positive controls and complementary techniques. Furthermore, we show that the *mito*-QC reporter can be multiplexed, especially if using spectral flow cytometry, to simultaneously study other cellular parameters such as viability or ROS production. Using this technique, we evaluated and characterized two prospective mitophagy inducers and further dissected their mechanism of action. Finally, using *mito*-QC reporter mice, we developed a protocol to measure mitophagy levels in the retina *ex vivo*. This novel methodology will propel mitophagy research forward and accelerate the discovery of novel mitophagy modulators.

## Introduction

Mitophagy is a subtype of selective autophagy that leads to the degradation and recycling of whole mitochondria. It can be further subdivided in two main pathways: PINK1/Parkin-dependent or receptor-mediated mitophagy. PINK1/Parkin-dependent mitophagy is traditionally triggered by loss of mitochondrial membrane potential (ΔΨm) (Narendra et al., 2010). The PINK1 kinase is recruited to the outer mitochondrial membrane (OMM) where, in homeostatic conditions, is translocated through the intermembrane space, processed by the peptidase MPP and sent back to the cytosol for degradation via proteasome (Hasson et al., 2013). Mitochondrial depolarization (loss of ΔΨm) uncouples PINK1 translocation, and it accumulates in the OMM. PINK1 then phosphorylates basally-ubiquitinated OMM proteins at Ubiquitin^Ser65^ and the analogous residue of Parkin, an E3 ligase that will further poly-ubiquitinate OMM proteins (Narendra et al., 2008). These events lead to a positive feedback loop that amplifies the targeting signals for mitophagy. Adaptor proteins with a ubiquitin-binding domain, such as CALCOCO2/NDP52, OPTN or SQSTM1/p62 (Lazarou et al., 2015), will recognize ubiquitinated proteins and recruit autophagy initiation machinery (Nguyen et al., 2016).

Receptor-mediated mitophagy is ubiquitin-independent and can also be triggered by hypoxia or developmental cues (Esteban-Martinez et al., 2017a). It is mediated by adaptor proteins that contain both a mitochondria-targeting sequence (MTS) and a LC3-interacting domain (LIR), namely BNIP3, BNIP3L/NIX, FUNDC1 or FKBP8 (Teresak et al., 2022). In recent years, it has been identified that other mechanisms such as cardiolipin externalization or changes in OMM lipid composition can also lead to direct recognition by LC3 (Teresak et al., 2022). Nonetheless, all pathways lead to mitochondria being engulfed into an autophagosome, which will later fuse with a lysosome to ensure degradation of its cargo.

Our lab has previously described a method to assess mitophagic degradation by flow cytometry using MitoTracker DeepRed (Mauro-Lizcano et al., 2015; Esteban-Martinez et al., 2017b). Even though it has been widely used and enables analysis of mitophagy flux, it still has some limitations. For example, a simultaneous induction of mitochondrial biogenesis might mask small but robust increases on mitophagy and use of lysosomal inhibitors is required (Esteban-Martinez et al., 2017b). The development of tandem fluorescent reporters has changed the way we analyze autophagy, as it allows us to monitor effective degradation inside acidic lysosomes using a combination of pH-sensitive (GFP, Keima) and pH-insensitive (mCherry) fluorescent proteins fused to a targeting sequence. Different mitophagy reporters such as MitoTimer, mtKeima or *mito*-QC have been developed, the latter also allowing fixation for downstream immunocytochemistry analysis (Jiménez-Loygorri et al., 2023; Jimenez-Loygorri and Boya, 2024).

**FIGURE 1:**
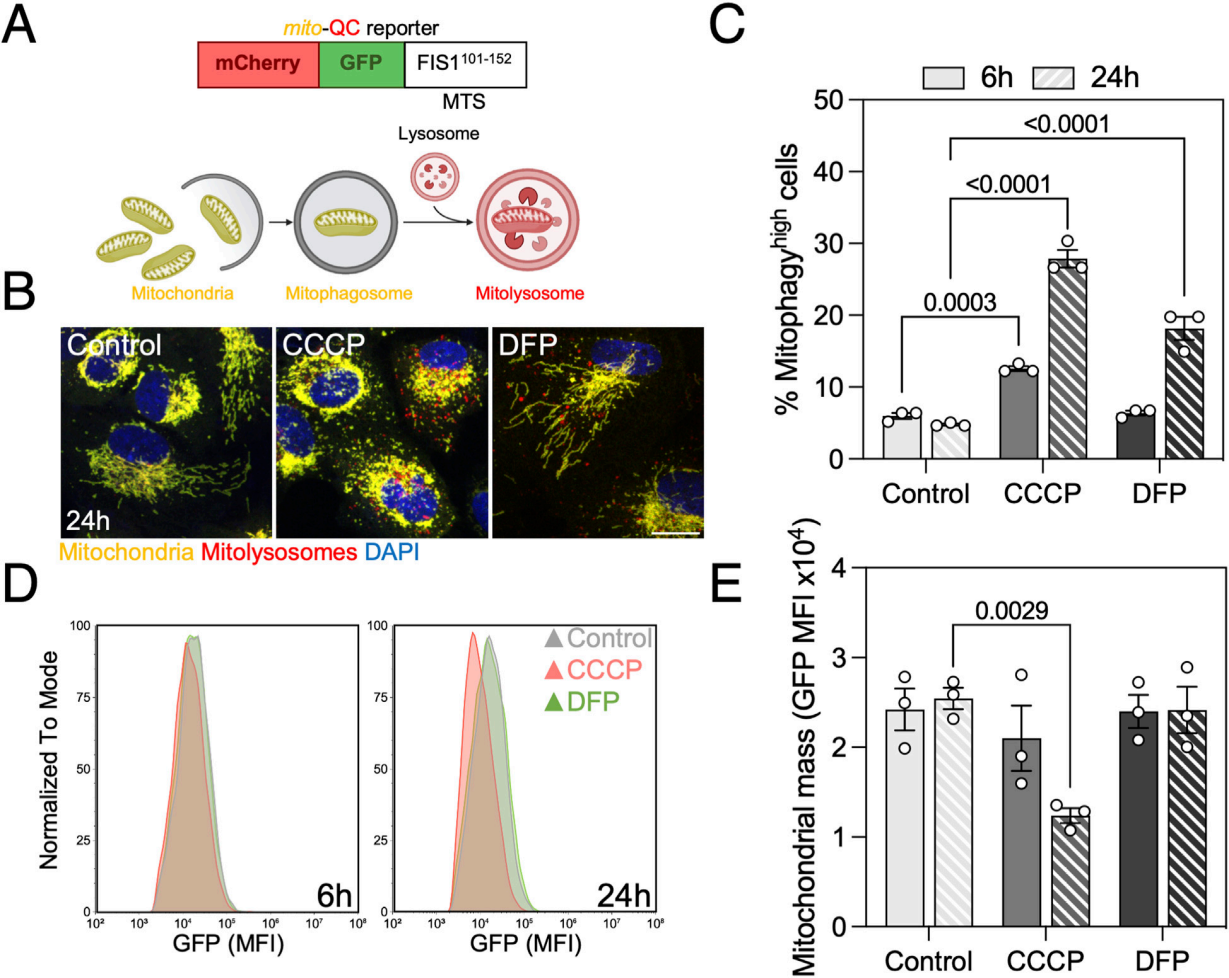
Mitophagy assessment in ARPE-19 cells by traditional flow cytometry using the *mito*-QC reporter. **(A)** Basis of the *mito*-QC reporter, whereby mitochondria are tagged with the chimeric protein mCherry-GFP-FIS1_101–152_. Upon mitophagy induction, mitochondria will be delivered to the lysosome where acidic pH will quench GFP and mitolysosomes will be identified as mCherry-only puncta. **(B)** ARPE-19 cells stably expressing the *mito*-QC reporter treated with 25 μM CCCP or 1 mM DFP for the indicated timepoints. **(C)** Quantification of % of mitophagy^high^ population as defined in Supplementary Figure S1. **(D)** Representative histogram of FITC-GFP mean fluorescence intensity (MFI), equivalent to mitochondrial mass, in cells from **(C)**. **(E)** Quantification of mitochondrial mass as shown in **(D)**. Scale bar, 15 μm. All data are expressed as the mean ± s.e.m. Dots represent independent experiments. *P* values were calculated using two-way ANOVA with Dunnett’s *post hoc* test. Diagrams were created using BioRender.

The *mito*-QC reporter consists of a fusion protein containing mCherry-GFP-FIS1^101−152^, that targets the OMM. In steady-state conditions, both mCherry and GFP fluoresce in the membrane of healthy mitochondria (Figure 1A) (McWilliams et al., 2018). Upon delivery of mitochondria to the lysosomes for degradation, acidic pH quenches GFP and mitolysosomes can be identified as mCherry-only puncta (Figure 1A). We have previously described the use of *mito*-QC to assess mitophagy levels by confocal immunofluorescence (Rosignol et al., 2020; Figueiredo-Pereira et al., 2023; Jimenez-Loygorri and Boya, 2024), however this procedure is time- and resource-consuming. In the present manuscript, we describe a medium-throughput protocol to assess simultaneously mitophagy and mitochondrial mass by flow cytometry *in vitro* and in *ex vivo* retinal cultures and highlight the possibility of performing more complex assays and targeted screens by multiplexing with additional fluorescent probes.

## Materials and methods

Herein, we present an optimized traditional and spectral flow cytometry protocol that provides sensitive simultaneous readouts of mitophagy and mitochondrial mass using tandem fluorescent reporters like *mito-*QC. This assay can be performed *in vitro* or using organotypic *ex vivo* culture and may be multiplexed to analyze other parameters such as reactive oxygen species (ROS) production or viability.

### Cell lines

ARPE-19 cells (Dunn et al., 1996) stably expressing the *mito-*QC reporter were generated by retroviral transfection in the laboratory of Dr. Ian G. Ganley as previously described (Montava-Garriga et al., 2020) and maintained in DMEM: F-12 medium (Gibco, 41966029, 21765037) supplemented with 15% FBS (Merck, F7524), 2 mM L-glutamine (Gibco, 25030081), and 1 U/mL Pen/Strep antibiotics (Gibco, 15140122) in a humidified incubator at 37°C, 5% CO_2_. To ensure stable expression of the reporter, selection was performed by adding 800 ug/mL Hygromycin B (Gibco, 10453982) after every passage.

### Seeding and treatments

For flow cytometry or immunofluorescence experiments, 5-6 × 10^4^ cells per well were seeded in a 24-well plate the day before the experiment and left to adhere overnight. For western blot analysis, 3 × 10^5^ cells were seeded in a 6-well plate and left to adhere overnight. Treatments were added for the indicated timepoints at the following concentrations: 25 μM CCCP (Carbonyl cyanide m-chlorophenyl hydrazone; 25 mM stock in DMSO; Merck, C2759), 1 mM DFP (Deferiprone; 125 mM stock in sterile H_2_O; Merck, 379409), 10 μM Fisetin (10 mM stock in EtOH; Merck, F4043), 50 μM Phenanthroline (50 mM stock in DMSO; Merck, 131377), 20 mM SI (Sodium iodate; 333 mM fresh stock in DMEM; Merck, S4007), 750 μM H_2_O_2_ (30% stock in DMEM; Fluka, 95300).

### Cell sample preparation

1. Medium containing floating dead cells was collected in a standard plastic flow cytometry tube.
2. Cells were washed with 500 μL of sterile PBS 1X to remove FBS residue and PBS was also collected.
3. 150 μL of 0.05% Trypsin (Gibco, 11500636) was added per well and cells were incubated for 5′ in an incubator at 37°C and 5% CO_2_. Verify that cells have detached from the bottom of the well using a brightfield microscope.
4. 500 μL of FBS-containing complete medium were added to each well to inactivate Trypsin.
5. Cells were collected in the flow cytometry tube and pelleted by centrifugation at 1,000 *g* for 5′.
6. Supernatant was discarded and 100–200 μL of complete medium was added to each tube. For selected experiments, cells were incubated with 1 nM MitoTracker Deep Red (Invitrogen, M22426), 1 μM MitoSOX Red (Invitrogen, M36008), for 30′ min or 1:2,000 ViaDye Red (Cytek, R7-60008), CellROX Deep Red 5 μM (Invitrogen, C10433) for 15′ at 37°C and 5% CO_2_.
7. Samples were resuspended by gentle shaking and, if necessary, 50 μL of 5X DAPI (4′,6-diamidino-2-phenylindole; Merck, D9542) were added to each tube, reaching a final concentration of 1 μg/mL, for viable population selection. If required, e.g. working with UV-autofluorescent compounds, there are far red-emitting nuclear dyes available such as TO-PRO-3 (Thermo Fisher, T3605).
8. Tubes were kept in ice until flow cytometry analysis.

### Retina dissection and *ex vivo* culture

C57BL/6J mice expressing the *mito-*QC reporter ubiquitously were generated and provided by Dr. Ian G. Ganley (McWilliams et al., 2018). Organotypic *ex vivo* retina culture was performed as previously described (Gomez-Sintes et al., 2017). Briefly, mice were sacrificed by cervical dislocation and both eyes were enucleated using curved forceps. Using Vannas scissors, a circular incision was made along the limbus and both cornea and lens were removed using fine forceps. The optic nerve was sectioned and the neuroretina was isolated by gently pulling from the RPE/choroid-containing eyecup. Retinas were cultured in flotation in a 24-well plate and maintained in DMEM supplemented with 1 μM Insulin (Merck, I2643), 2 mM L-Glutamine, 100 U/mL penicillin and 0.1 mg/mL streptomycin. Treatments were added for the indicated timepoints at the following concentrations: 25 μM CCCP (25 mM stock in DMSO), 1 mM DFP (125 mM stock in sterile H_2_O).

### *Ex vivo* retina sample preparation

1. Similarly, medium containing floating dead cells was collected in a standard plastic flow cytometry tube.
2. Whole retinas were washed with 500 μL of sterile PBS 1X to remove medium residue and PBS was also collected.
3. 300 μL of 5 mg/mL Trypsin in HBSS (Gibco, 14170-088) was added per well and retinas were incubated for 5–10′ in an incubator at 37°C and 5% CO_2_.
4. 900 μL of 10% FBS in HBSS were added to each well to inactivate Trypsin and retinas were dissociated by gentle pipetting using a p1000 tip (10–20 times).
5. Single cell suspension was collected in the flow cytometry tube and pelleted by centrifugation at 1,000 *g* for 5′.
6. Supernatant was discarded and 200 μL of complete medium was added to each tube.
7. Samples were resuspended by gentle shaking and 50 μL of 5X DAPI was added to each tube, reaching a final concentration of 1 μg/mL, for viable population selection.
8. Tubes were kept in ice until flow cytometry analysis.

### Conventional flow cytometer setup and gating

Using a CytoFLEX S V4-B2-Y4-R3 Flow Cytometer (Beckman Coulter), at least 10,000 events were acquired using mCherry-PI (610/20), FITC-GFP (525/40), PB450-DAPI (450/45) and APC (660/10) emission filters. Gating of the viable cell population was performed as shown in Supplementary Figures S1, S3. Control cells were used to set the threshold of mitophagy^high^ population defined by a mCherry/GFP ratio of ∼5%. The percentage of DAPI^−^ cells, mitophagy^high^ cells, FITC-GFP MFI and APC MFI were exported for downstream analysis performed using CytExpert v1.2 (Beckman Coulter).

### Spectral flow cytometer setup and gating

Using a Cytek Aurora equipped with five lasers (Cytek Biosciences), at least 10,000 events were acquired. Similarly, control cells were used to set the threshold of mitophagy^high^ population defined by a mCherry/GFP ratio of ∼5%. Wild-type, control cells and unstained cells were used to set the gates and spectra for every probe (Supplementary Figure S5). Analysis was performed using SpectroFlo (Cytek Biosciences) for raw data unmixing and downstream analysis was performed using Flowjo v10.10 (BD Biosciences).

### Immunofluorescence

ARPE-19 were seeded on glass coverslips, treated as indicated and fixed using 3.7% PFA containing 175 mM HEPES (Gibco, 15630) at pH 7.0 for 15 min. Cells were incubated with 1 µg/mL DAPI in PBS pH 7.0 for 15 min, washed 3 times with PBS pH 7.0 and mounted using or ProLong Diamond (Thermo Fisher, P36961). Images were captured with a 0.5-µm z-step using a Leica TCS SP8 confocal microscope equipped with a × 63 immersion objective.

### Protein isolation, quantification and western blot

Adherent cells were scraped in cold RIPA lysis buffer (Merck, R0278) supplemented with protease and phosphatase inhibitors. Protein concentration was determined using the Pierce BCA Protein Assay (Thermo Fisher, 23225) following the manufacturer’s instructions. Total protein extract (15–30 µg) was supplemented with 4X Laemmli loading buffer (Bio-Rad, 1610747) and resolved using Any kD Criterion TGX Precast Stain-free gels (Bio-Rad, 5678124). Proteins were transferred to 0.2 µm PVDF membranes using a TransBlot Turbo Transfer System (Bio-Rad) and total protein was quantified using Ponceau S staining (Merck, 78376). Membranes were blocked with 5% non-fat milk in TBS-T (0.5% Tween-20 (Bio-Rad, 1706531) in PBS) for 1 h. Membranes were then incubated overnight at 4°C in primary antibodies diluted 1:1,000 (Supplementary Table S1) in 5% BSA in TBS, and subsequently for 1 h at room temperature in secondary antibodies diluted 1:2,000 in TBS-T (Supplementary Table S1). Membranes were developed using Pierce ECL Western Blotting substrate (Thermo Fisher, 32106) or Amersham ECL Prime (Cytiva, 10308449) and x-ray film (AGFA) using a CURIX 60 Processor (AGFA).

### Statistical analysis

Data shown in figures represents the mean ± s.e.m. of at least three independent experiments with biological replicates. Differences between groups were assessed using Student’s *t*-test (two groups), one-way or two-way ANOVA (more than two groups) with appropriate *post hoc* tests. A *P*-value under 0.05 was considered statistically significant and exact, corrected *P*-values are shown. Raw data management was done in Microsoft Excel and statistical analyses were performed using GraphPad Prism 10.0 software.

## Results

### *Mito*-QC reporter provides sensitive detection of mitophagy and mitochondrial mass *in vitro* and *ex vivo*

The ARPE-19 cell line originated from the retinal pigment epithelium of a healthy adult donor, and underwent spontaneous immortalization (Dunn et al., 1996). ARPE-19 cells stably expressing the *mito*-QC reporter were used for all experiments (Montava-Garriga et al., 2020) and mitophagy levels were defined as the population of cells with an increased mCherry/GFP ratio (Supplementary Figure S1).

We treated *Park2-*expressing ARPE-19 cells with CCCP and DFP to validate the sensitivity of the reporter when analyzed by flow cytometry. CCCP is a protonophore that disrupts ΔΨm by uncoupling the proton gradient, and is traditionally used as an inducer of PINK1/Parkin-mediated mitophagy (Narendra et al., 2008). DFP is an iron chelator that inhibits HIF-prolyl hydroxylases (PHDs), leading to HIF-1α stabilization, hypoxia-like response and upregulation of downstream mitophagy regulators such as *BNIP3* or *BNIP3L*/NIX that engage in receptor-mediated mitophagy (Allen et al., 2013). Both compounds induced mitophagy at the 24 h timepoint, but only CCCP induced a significant increase after 6 h (Figures 1B, C).

Simultaneously, this reporter allows for quantification of mitochondrial mass defined by the mean fluorescence intensity (MFI) of its GFP component. CCCP significantly decreased cytosolic mitochondrial mass at both timepoints, but no changes were observed with DFP (Figures 1D, E). These results point to either a possible compensation by mitochondrial biogenesis to maintain homeostasis during receptor-mediated mitophagy or, since this pathway requires transcriptional activity to increase BNIP3 and BNIP3L/NIX levels, that mitochondrial mass decrease will be observed at a later timepoint. In parallel, we performed a comparative analysis using spectral flow cytometry and obtained similar results regarding mitophagy levels (Supplementary Figure S2A) and mitochondrial mass (Supplementary Figure S2B) after mitophagy induction with CCCP and DFP, indicating that *mito*-QC can also be measured using this novel technology.

**FIGURE 2:**
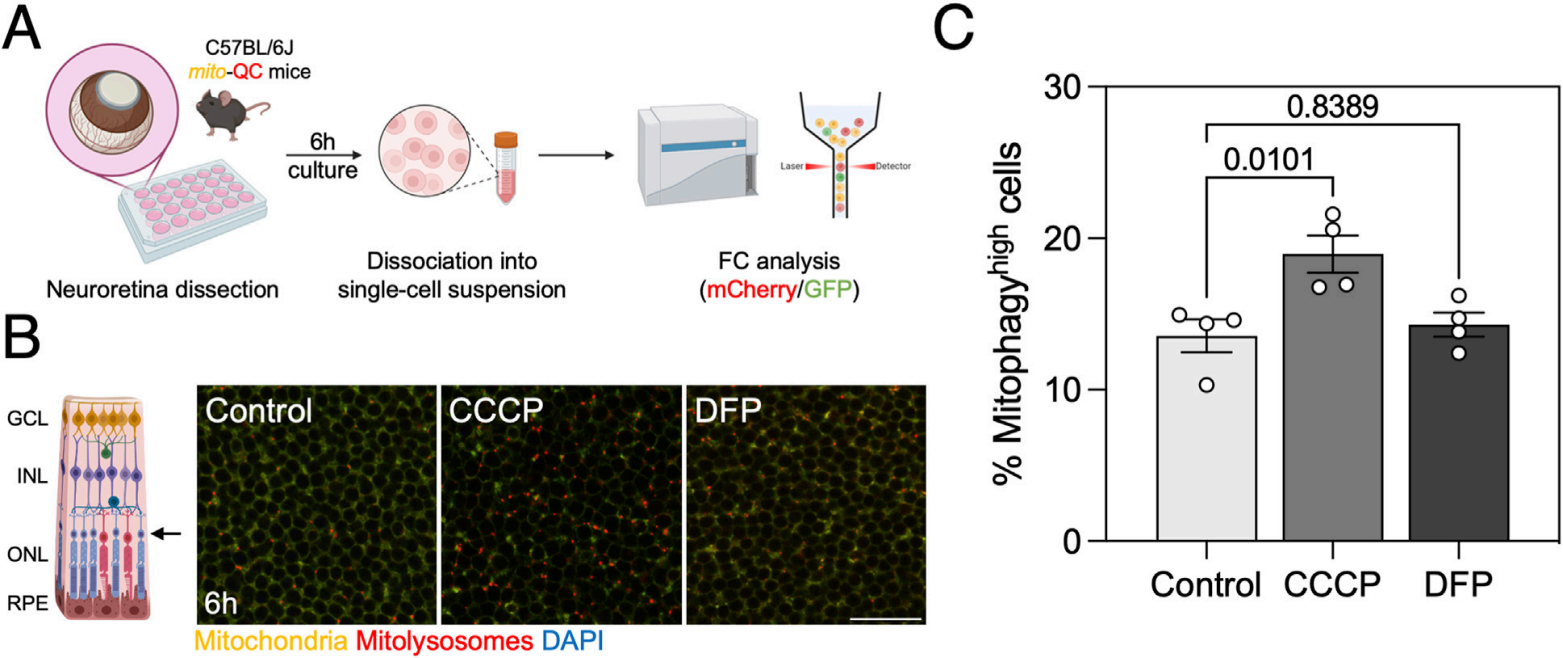
Mitophagy assessment in organotypic *ex vivo* retina culture. **(A)** Neuroretinas from C57BL/6J mice expressing the *mito*-QC reporter were dissected and cultured *ex vivo* for 6 h in defined medium in the present of 25 μM CCCP or 1 mM DFP. Samples were dissociated into a single-cell suspension and analyzed by FC. **(B)** Representative images of the photoreceptor-containing outer nuclear layer (ONL). **(C)** Quantification of the % of mitophagy^high^ cells in neuroretina *ex vivo* culture as defined in Supplementary Figure S3. Scale bar, 25 μm. All data are expressed as the mean ± s.e.m. Dots represent individual mice. *P* values were calculated using one-way ANOVA with Dunnet’s *post hoc* test. Diagrams were created using BioRender.

In addition, we also analyzed mitophagy in organotypic *ex vivo* retina culture from *mito*-QC mice (Figure 2A; Supplementary Figure S3). Replicating our findings *in vitro*, CCCP was able to induce mitophagy after 6 h of treatment but DFP was not (Figures 2B, C). The *mito*-QC reporter can therefore be used as a medium-throughput readout to assess mitophagy and mitochondrial mass *in vitro* and *ex vivo*.

### Fisetin and phenanthroline induce mitophagy but no mitochondrial biogenesis

**FIGURE 3:**
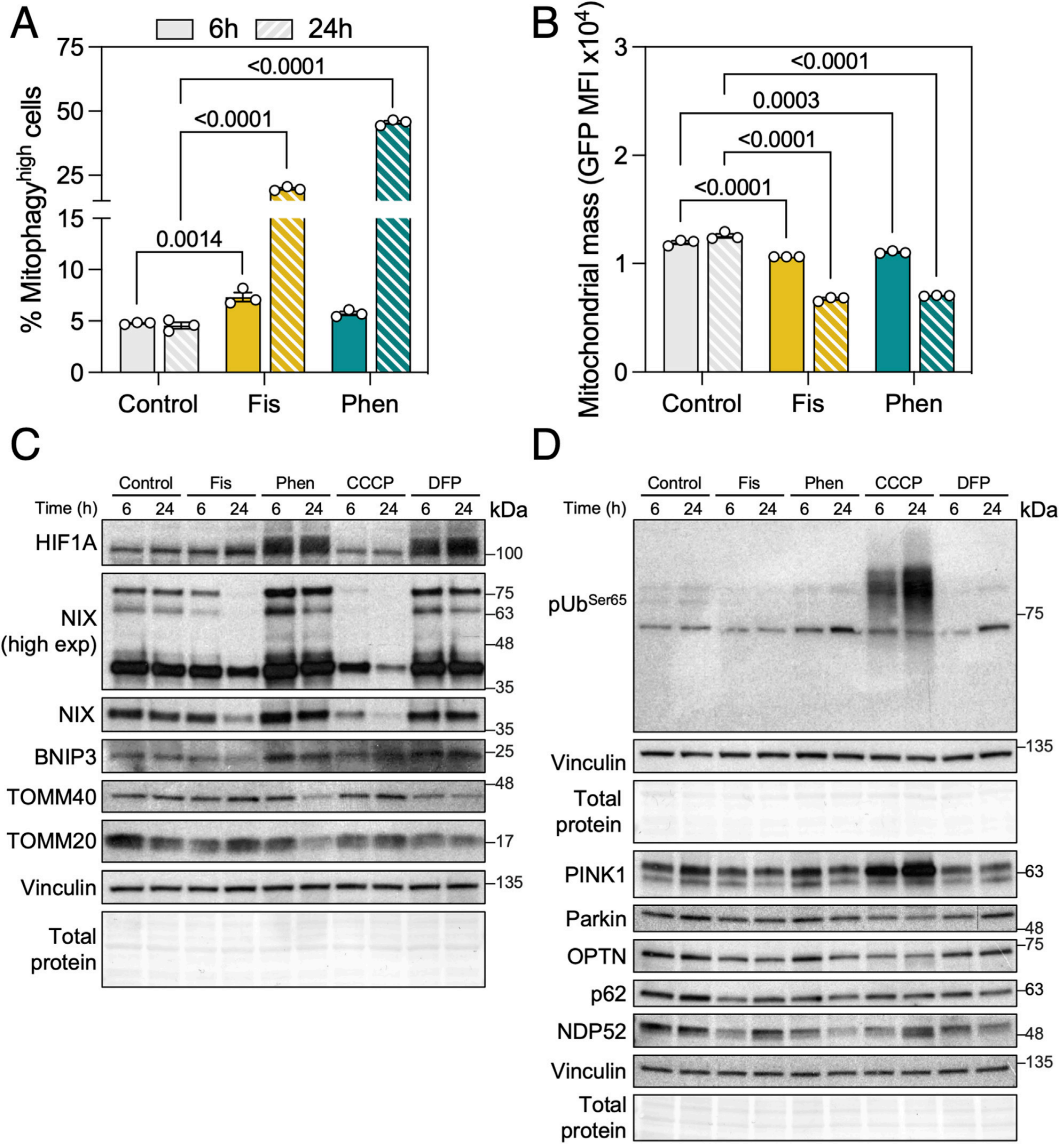
Phenanthroline and Fisetin are potent mitophagy inducers. **(A)** ARPE-19 *mito*-QC cells were treated with 10 μM Fisetin (Fis) or 50 μM Phenanthroline (Phen) for the indicated timepoints, quantification of % of mitophagy^high^ population is shown. **(B)** Quantification of mitochondrial mass (GFP MFI). **(C)** Immunobloting against proteins involved in receptor-mediated mitophagy (HIF-1α, BNIP3L/NIX, BNIP3) and mitochondrial markers (TOMM20, TOMM40). Vinculin was used as a loading control. **(D)** Immunoblotting against proteins involved in PINK1/Parkin-dependent mitophagy (phospho-Ubiquitin^Ser65^, PINK1, Parkin, OPTN, SQSTM1/p62, NDP52). Vinculin was used as a loading control. All data are expressed as the mean ± s.e.m. Dots represent independent experiments. *P* values were calculated using two-way ANOVA with Dunnett’s *post hoc* test.

To further validate the use of the *mito*-QC reporter, we tested two compounds that we had previously found to significantly increase mitophagy using MitoTracker DeepRed flux assay: Fisetin and Phenanthroline (Mauro-Lizcano et al., 2015). Both compounds induced a robust mitophagy increase at 6 h that was exacerbated by 24 h (Figure 3A). With a similar kinetic to CCCP, both compounds significantly reduced mitochondrial mass at both timepoints analyzed suggesting no concomitant activation of mitochondrial biogenesis (Figure 3B). To further dissect the mechanism of action of these mitophagy inducers, we performed immunoblotting against the mediators of iron depletion-induced mitophagy and observed that Phenanthroline increased the protein levels of HIF-1α and its downstream targets, the mitophagy receptors BNIP3 and BNIP3L/NIX (Figure 3C). Fisetin decreased BNIP3L/NIX levels at the 24 h timepoint, but this observation might rather be representative of acute mitophagy as BNIP3L/NIX is a resident protein at the OMM (Wilhelm et al., 2022). We also evaluated PINK1 stabilization, phospho-Ubiquitin^Ser65^ and ubiquitin adaptor (OPTN, SQSTM1/p62, CALCOCO2/NDP52) levels but observed no differences with Fisetin nor Phenanthroline treatment (Figure 3D), indicating that their activity is PINK1/Parkin-independent.

### Mitophagy is impaired in an *in vitro* model of retinal degeneration

**FIGURE 4:**
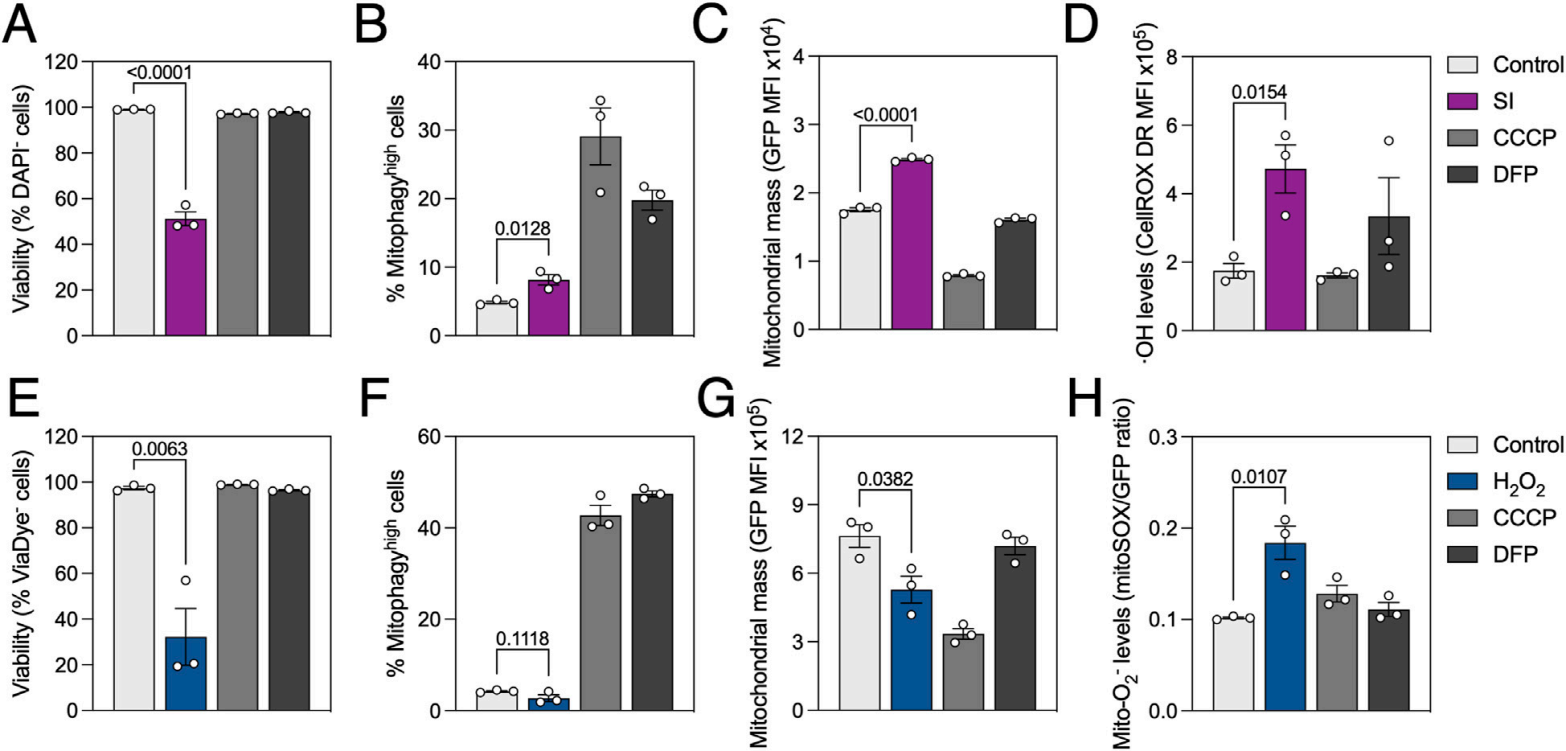
Oxidative stress reduces cell viability, differentially modulates mitophagy and stimulates ROS production. ARPE-19 *mito*-QC cells were treated with 20 mM sodium iodate (SI) or 750 μM H_2_O_2_ for 24 h. **(A)** Simultaneous measurement of viability by nuclear exclusion assay using DAPI in SI-treated cells. Viable population does not incorporate the dye and are identified as DAPI^−^. **(B)** Quantification of % of mitophagy^high^ population in SI-treated cells. **(C)** Quantification of mitochondrial mass (GFP MFI) in SI-treated cells. **(D)** Simultaneous measurement of reactive oxygen species (ROS) using CellROX Deep Red dye in SI-treated cells. **(E)** Simultaneous measurement of viability using ViaDye Red in H_2_O_2_-treated cells. Viable population does not incorporate the dye and are identified as ViaDye^−^. **(F)** Quantification of % of mitophagy^high^ population in H_2_O_2_-treated cells. **(G)** Quantification of mitochondrial mass (GFP MFI) in H_2_O_2_-treated cells. **(H)** Simultaneous measurement of mitochondrial ROS using MitoSOX Red dye in H_2_O_2_-treated cells. All data are expressed as the mean ± s.e.m. Dots represent independent experiments. *P* values were calculated using one-way ANOVA with Dunnet’s *post hoc* test.

We previously described high mitophagy levels in the neuroretina and RPE (McWilliams et al., 2019; Jimenez-Loygorri et al., 2024). Sodium iodate (SI) is commonly used as a pharmacological model of age-related macular degeneration (AMD), both *in vitro* and *in vivo* (Chowers et al., 2017). Even though AMD has been historically linked to mitochondrial dysfunction (Fisher and Ferrington, 2018) and impaired autophagy (Kaarniranta et al., 2023), evidence on the role of mitophagy in AMD progression is scarcer (Jiménez-Loygorri et al., 2023). Using *mito*-QC analysis by flow cytometry we observed that treatment with SI for 24 h slightly increased mitophagy levels (Figure 4A) concomitant with a moderate but significant increase in mitochondrial mass (Figure 4B). Traditional flow cytometers additionally equipped with violet and red lasers allow for multiplexing with additional dyes. We combined *mito-*QC readout with DAPI (λ_ex_ = 350 nm; λ_em_ = 465 nm), for nuclear exclusion viability assessment, and CellROX Deep Red (λ_ex_ = 644 nm; λ_em_ = 665 nm), a fluorogenic probe with high sensitivity for OH radical detection. SI decreased cell viability to ∼70% (Figure 4C) and stimulated ROS production (Figure 4D), in line with previously published data (Chan et al., 2019).

Taking advantage of spectral flow cytometry which can detect the whole emission spectrum under different excitation lasers and dissect the contribution of every fluorophore, we also combined *mito*-QC with traditional red-emitting fluorescent probes. Treatment with H_2_O_2_ significantly decreased viability to ∼40%, measured using ViaDye Red (λ_ex_ = 615 nm; λ_em_ = 740 nm) which binds to intracellular proteins when the plasma membrane is compromised (Figure 4E). While no changes were observed regarding mitophagy levels (Figure 4F), mitochondrial mass was decreased in H_2_O_2_-treated cells (Figure 4G). These observations were concurrent with increased mitochondrial O_2_^−^ production, measured using MitoSOX Red dye (λ_ex_ = 396 nm; λ_em_ = 610 nm; Figure 4H). Markedly, all results were replicated in wild-type ARPE-19 cells using the same dyes (Supplementary Figures S4A, C) and MitoTracker Deep Red (λ_ex_ = 644 nm; λ_em_ = 665 nm) as a surrogate to measure mitochondrial mass (Supplementary Figure S4B). Spectral unmixing overcomes partial spectrum overlap and allows for simultaneous measurement of *mito*-QC and probes with similar fluorescence profiles.

## Discussion

In the present manuscript we provide a standardized protocol to assess mitophagy in cells and *ex vivo* dissociated tissue using the tandem fluorescent *mito*-QC reporter via flow cytometry. Our results with *mito*-QC validate the ability of CCCP and Fisetin to stimulate mitophagy flux, as previously reported by our group using the MitoTracker Deep Red approach (Esteban-Martinez et al., 2017b), and now show that Phenanthroline also induces mitophagy in ARPE-19 cells. Finally, we highlight the possibility of simultaneously measuring other mitochondrial or intracellular parameters using additional probes and conventional or spectral flow cytometry. We also report that mitophagy is impaired in cell type-relevant oxidative stress models (SI, H_2_O_2_). Furthermore, the use of *mito*-QC bypasses the need for lysosomal degradation inhibitors (Mauro-Lizcano et al., 2015) and the putative confounding effects of drug or ROS interaction MitoTracker dye (Xiao et al., 2016).

The analysis of *mito*-QC by flow cytometry has been crucial for key findings such as the metabolic readaptation following iron chelation (Long et al., 2022) or to understand the interplay between BNIP3L/NIX-mediated mitophagy and pexophagy (Wilhelm et al., 2022). While protocols to measure mitophagy by flow cytometry using mt-Keima are readily available (Um et al., 2018; Winsor et al., 2020), this is, to our knowledge, the first protocol focused on the next-generation fixable *mito*-QC reporter. Compared to mt-Keima, the possibility of fixing *mito*-QC opens the possibility of implementing, for example, flow cytometry immunophenotyping strategies to assess mitophagy in specific cell subsets within a heterogenous sample. A novel tandem reporter called SRAI (Signal-Retaining Autophagy Indicator) composed of a pH-insensitive CFP variant (TOLLES) and a highly pH-sensitive YFP variant (YPet) has recently been developed and utilized to measure both mitophagy (Katayama et al., 2020) and ER-phagy (Jimenez-Moreno et al., 2023) *in vitro* or using viral vector delivery *in vivo*. There are also published guidelines on how to measure mitochondrial turnover using MitoTimer (Hernandez et al., 2013), but interpretation of data using MitoTimer should be cautious as it does not directly report mitochondria degradation within lysosomes (Gottlieb and Stotland, 2015).

While previous reports have raised concerns regarding the evaluation of PINK1/Parkin-dependent mitophagy using the *mito*-QC reporter (Liu et al., 2021), our results show that CCCP induced detectable levels of mitophagy as early as 6 h in ARPE-19 *mito*-QC cells. Similarly, we have previously reported an increase in mCherry^+^GFP^−^ mitolysosomes in response to Antimycin + Olygomycin (AO), another classical inducer of the PINK1/Parkin-dependent mitophagy pathway (Rosignol et al., 2020).

Phenanthroline is a metal ion chelator commonly used as a ligand in the chemical industry (Park et al., 2012). It had previously been suggested that phenanthroline induces severe DNM1L/DRP1-dependent mitochondrial fragmentation that leads to mitophagy (Park et al., 2012). We have now further characterized its mechanism of action showing that phenanthroline induces HIF-1α stabilization and transcription of downstream mitophagy receptors BNIP3L/NIX and BNIP3. Interestingly, in our previous work we found that phenanthroline was not able to induce mitophagy in neuroblastoma-derived SH-SY5Y cells (Mauro-Lizcano et al., 2015) but a 10-fold increase was observed in ARPE-19 cells, indicating that its effect might be cell type-dependent. Phenanthroline has also been proposed as a pro-survival agent against apoptosis (Maitra et al., 2021) and parthanatos-mediated cell death (Chiu et al., 2012).

Fisetin is a natural flavonoid that acts as a potent SIRT1 NAD^+^-dependent histone deacetylase activator (Jang et al., 2012). The NAD^+^/NADH ratio is affected by mitochondrial function, and *vice versa* (Lautrup et al., 2024). Fisetin has been described to increase the NAD^+^/NADH ratio and induce mitophagy (Jang et al., 2012), through a mechanism that was dependent on the ubiquitin adaptor SQSTM1/p62 (Molagoda et al., 2021). Despite a robust increase in mitophagy in SH-SY5Y (Mauro-Lizcano et al., 2015) and ARPE-19 cells, we did not observe any changes in traditional PINK1/Parkin-dependent or receptor-mediated mitophagy effectors. The mechanism of action of Fisetin remains to be elucidated, but it has shown neuroprotective effects in models of neuroinflammation involving NLRP3 inflammasome activation (Molagoda et al., 2021; Ding et al., 2022). Both compounds showed no signs of cytotoxicity *in vitro* and warrant further exploration in diseases involving impaired mitophagy.

Previous evidence in the literature was suggestive of impaired autophagy in the SI model of AMD-associated geographic atrophy (Chan et al., 2019). In the present manuscript we indeed observed a slight increase in mitophagy but also a concomitant accumulation of mitochondria in the viable population of cells challenged with SI, replicating our previous findings using *mito*-QC immunofluorescence (Jimenez-Loygorri et al., 2024). Primary RPE cultures from patients with AMD similarly display marked mitochondrial dysfunction (Ferrington et al., 2017) as well as autophagy defects (Ye et al., 2016) that could be a result of dysfunctional mitophagy. Boosting mitophagy could therefore be a novel potential therapeutic strategy in the prevention and/or treatment of AMD.

Finally, we also performed a comparison between widely available standard flow cytometry and spectral cytometry, exploring its added value. Polychromatic flow cytometry or standard flow cytometry is based on the principle one detector, one fluorochrome thanks to a series of dichroic filters. So, only a portion of the emitting signal can be collected. Spectral flow cytometry can collect the full fluorescence spectrum of every cell allowing the separation and differentiation among their spectral signatures, allowing more elaborate, multiplexed assays. This is possible thanks to the spectral unmixing algorithm which identifies the spectral signature of every fluorochrome plus the autofluorescence of the cells and resolves the complete spectra of every cell based on these parameters, allowing to differentiate the contribution of each fluorochrome in a certain wavelength (Robinson, 2022). Using this methodology, we were able to combine *mito*-QC analysis with green- and red-emitting intracellular probes that present partial spectral overlap with GFP and mCherry (Supplementary Figure S5).

Mitophagy analysis by flow cytometry, using *mito*-QC or similar reporters, therefore represents an update to previously used methodology, reducing time, cost and resources when compared to traditional microscopy analysis. Herein we provide a standardized protocol for *mito*-QC analysis using traditional and spectral flow cytometry, multiplexing with an array of intracellular probes and insight on mitophagy regulation using two inducers and models of oxidative stress.

## Summary blurb

*mito*-QC reporter analysis by flow cytometry is a reliable and semi-high throughput method to measure mitophagy *in vitro* and *ex vivo*.

## Data Availability

The raw data supporting the conclusions of this article will be made available by the authors, without undue reservation.

## Abbreviations

ΔΨm, Mitochondrial membrane potential; AMD, Age-related macular degeneration; BNIP3, BCL2/adenovirus E1B 19 kDa protein-interacting protein 3; BNIP3L/NIX, BCL2/adenovirus E1B 19 kDa protein-interacting protein 3-like; CCCP, Carbonyl cyanide m-chlorophenyl hydrazone; DFP, Deferiprone; FIS1, Mitochondrial fission 1 protein; GFP, Green fluorescent protein; HIF-1α, Hypoxia-inducible factor 1-alpha; MFI, Mean fluorescence intensity; PINK1, PTEN-induced kinase 1; OMM, Outer mitochondrial membrane; ROS, Reactive oxygen species; RPE, Retinal pigment epithelium; SI, Sodium iodate.
