# Supplementary material for: Fast and quantitative mitophagy assessment by flow cytometry using the mito-QC reporter
Source: Front Cell Dev Biol. 2024 Sep 11;12:1460061. doi: 10.3389/fcell.2024.1460061 (PMC11422238; doi:10.3389/fcell.2024.1460061)
Supplement: Supplementary file 1 [file DataSheet1.docx]

**Fast and quantitative mitophagy assessment by flow cytometry using the *mito*-QC reporter**

**Authors:** Juan Ignacio Jiménez-Loygorri^1^, Carlos Jiménez-García^2^, Álvaro Viedma-Poyatos^1^, Patricia Boya^1,2*^

**Affiliations:**

^1^Department of Cellular and Molecular Biology, Centro de Investigaciones Biológicas Margarita Salas, CSIC, Madrid, Spain

^2^Department of Neuroscience and Movement Science, Faculty of Science and Medicine, University of Fribourg, Switzerland

**Corresponding author: patricia.boya@unifr.ch*

**Supplementary Materials** including:

- Supplementary Figures 1 to 5
- Supplementary Table 1

**
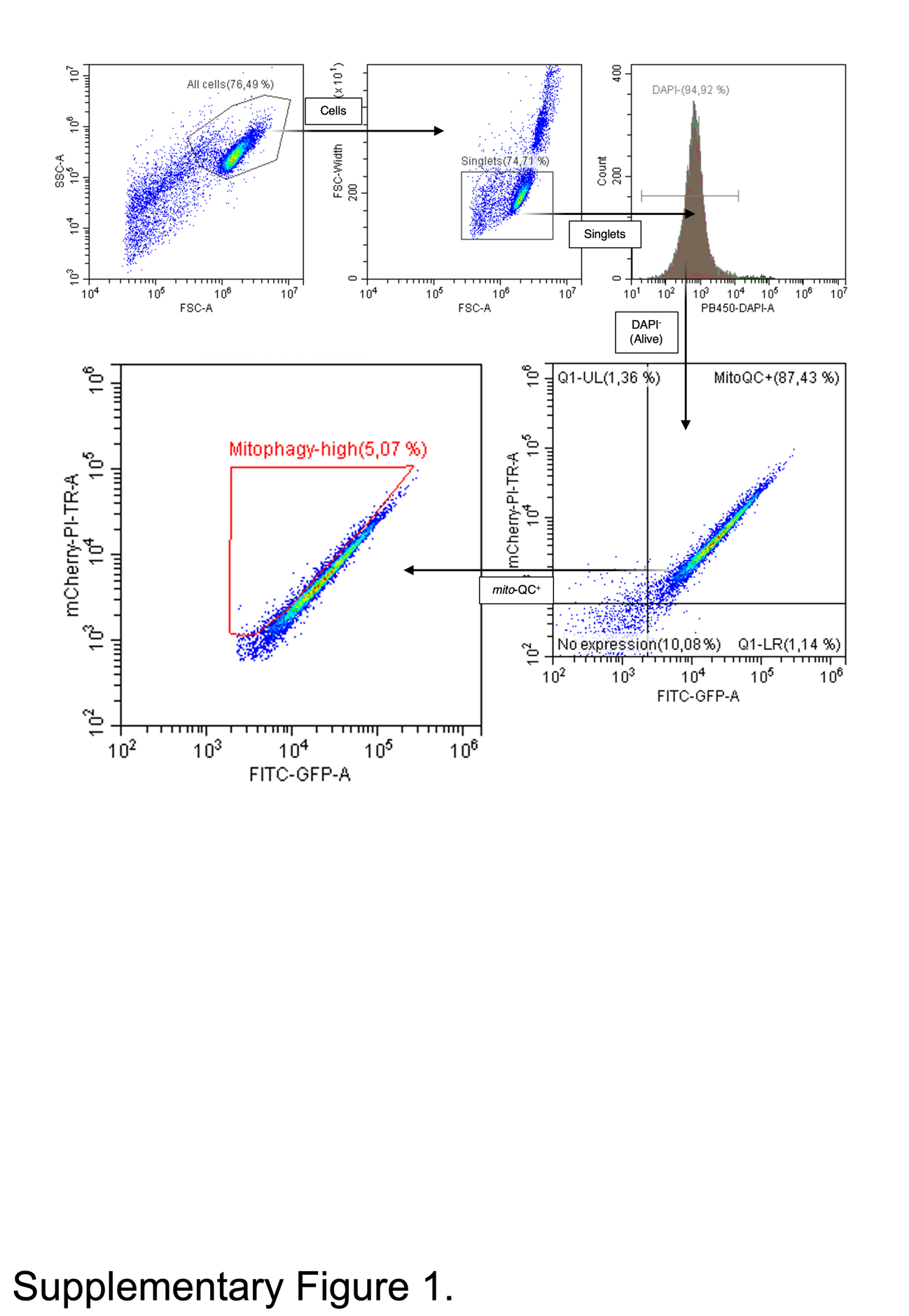
**

**Supplementary figure 1. Gating strategy for viable population and mitophagy analysis *in vitro*.** Doublet exclusion and viable cell population determination via membrane dye exclusion were used in all experiments.


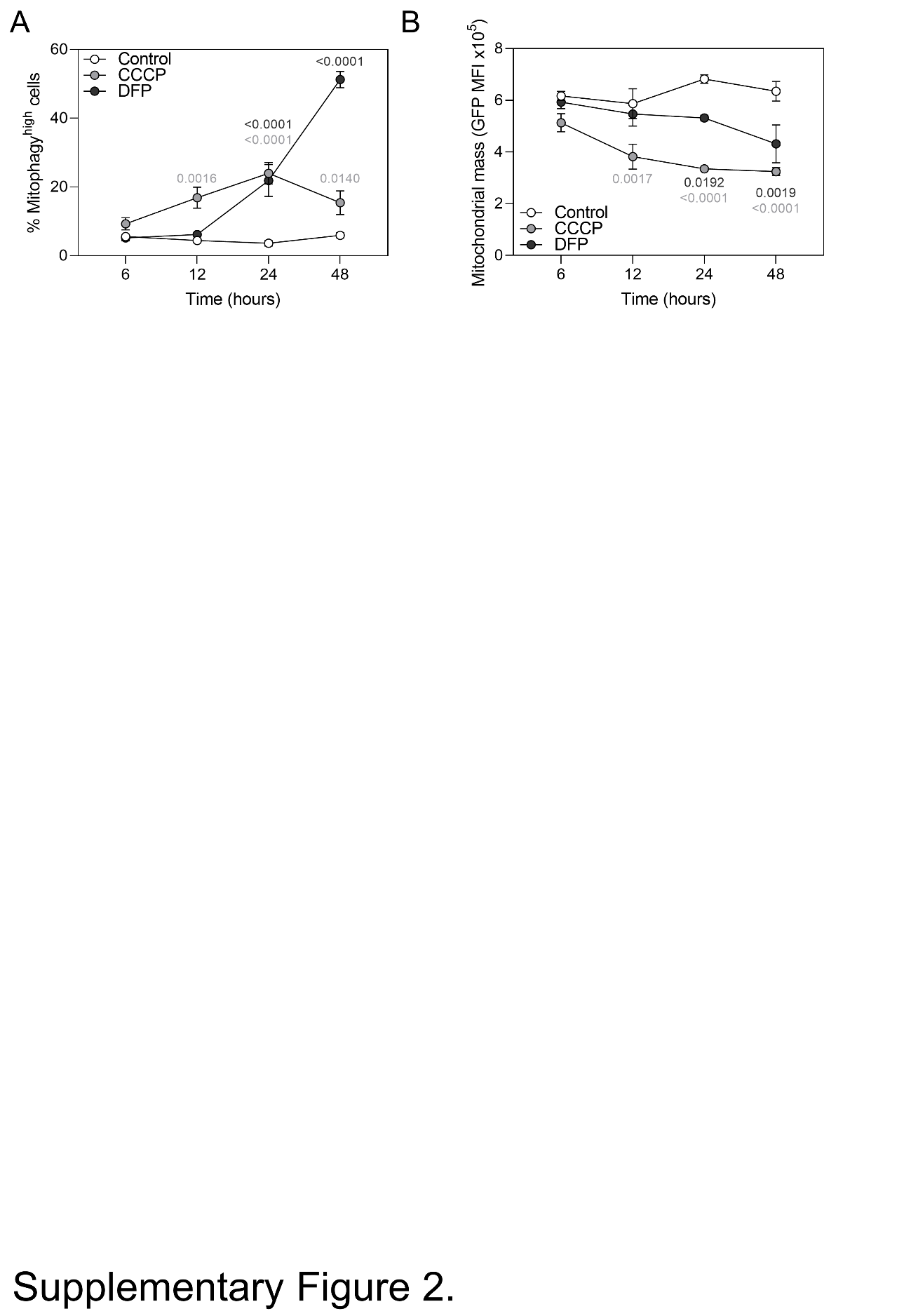


**Supplementary figure 2. Mitophagy assessment in ARPE-19 cells by spectral flow cytometry using the *mito*-QC reporter.** (**A**) ARPE-19 cells stably expressing the *mito*-QC reporter treated with 25 μM CCCP or 1 mM DFP for the indicated timepoints. Quantification of % of mitophagy^high^ population. (**B**) Quantification of mitochondrial mass as shown in **A**. All data are expressed as the mean ± s.e.m. Dots represent independent experiments. *P* values were calculated using two-way ANOVA with Dunnett’s *post-hoc* test.

**
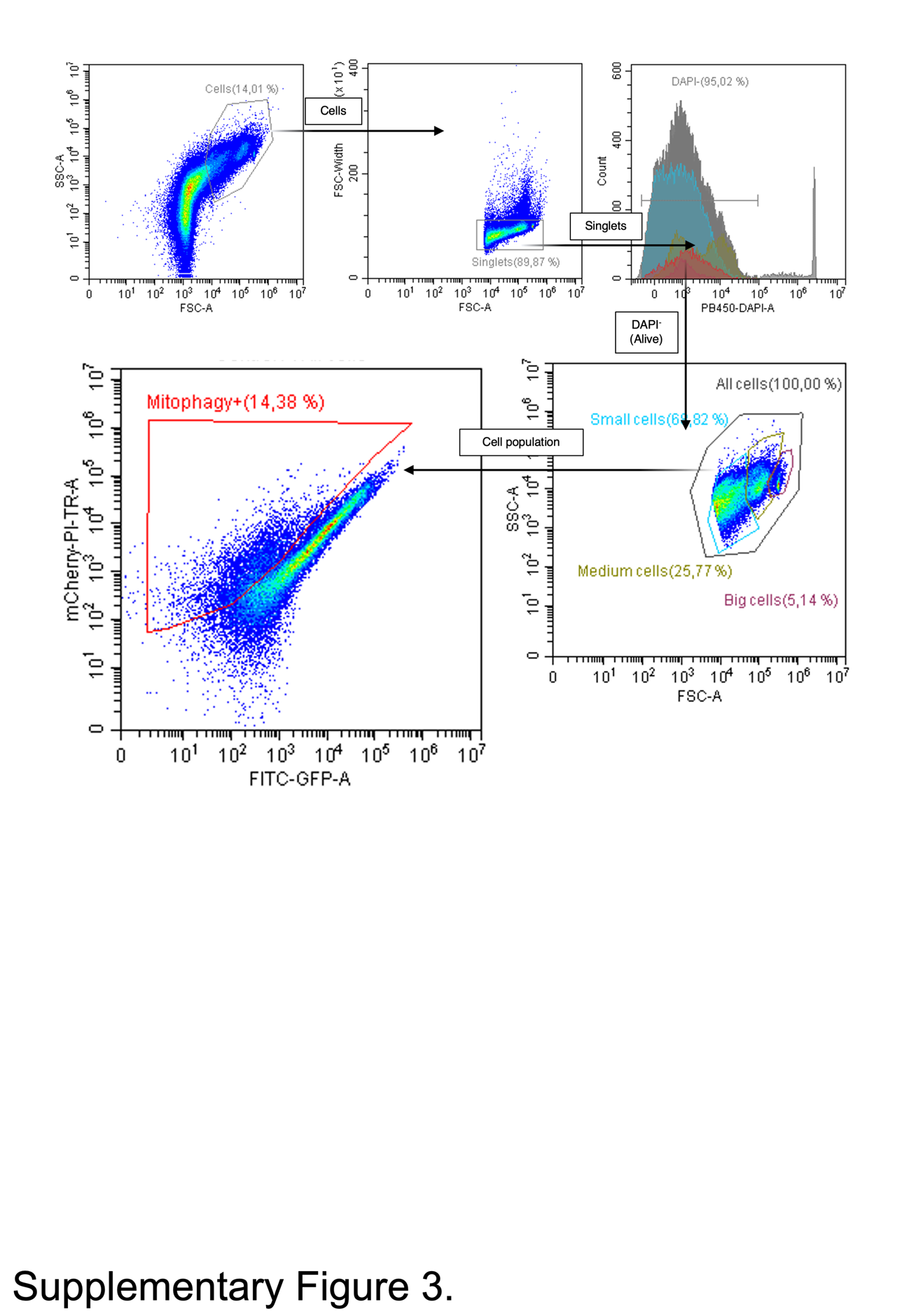
**

**Supplementary figure 3. Gating strategy for viable population and mitophagy analysis *ex vivo* neuroretina culture.** Doublet exclusion and viable cell population determination via nuclear dye exclusion were used in all experiments.

**
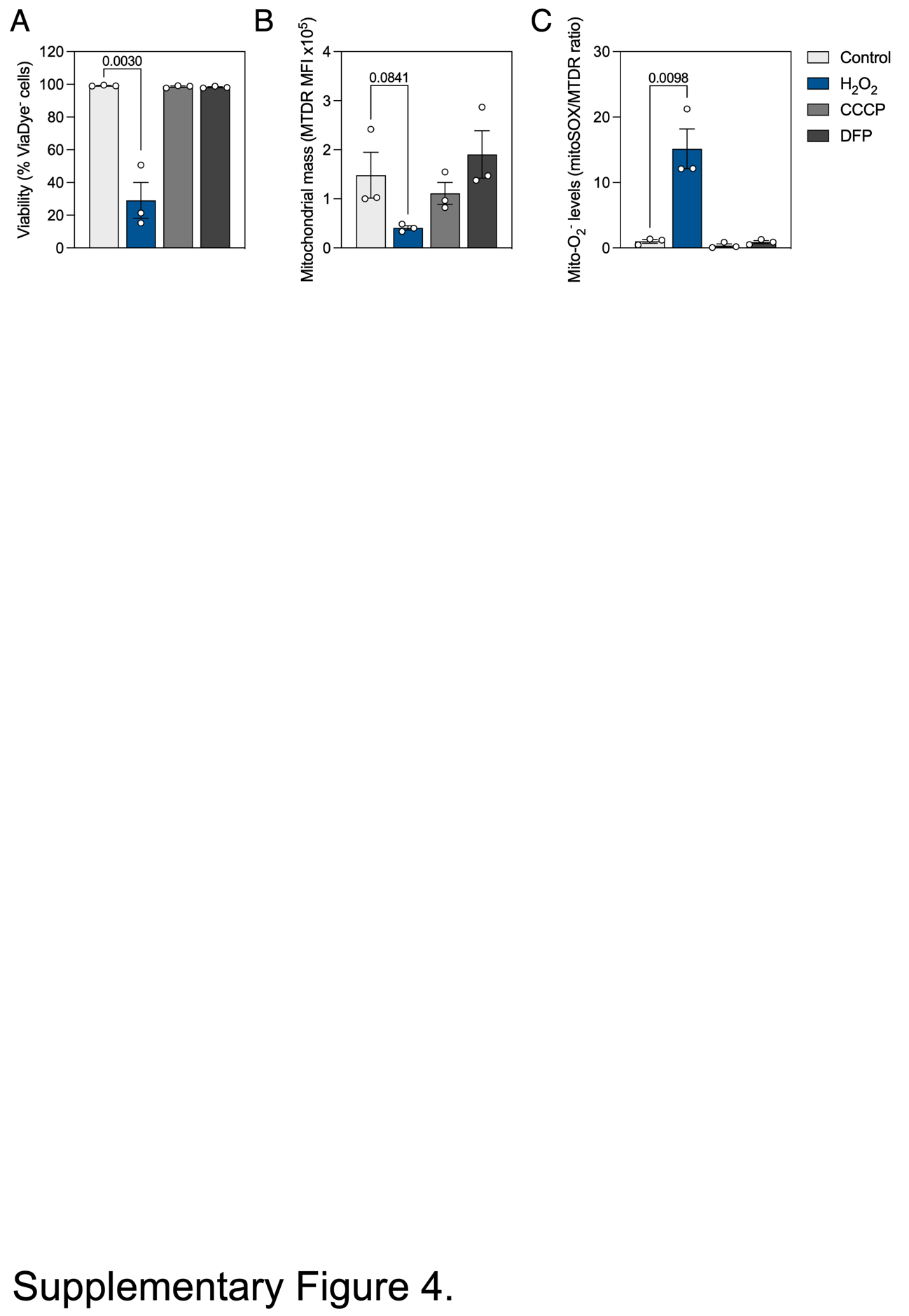
**

**Supplementary figure 4. H_2_O_2_ reduces cell viability, mitochondrial mass and stimulates ROS production in wild-type ARPE-19 cells.** ARPE-19 wild-type cells were treated with 750 μM H_2_O_2_ for 24 hours. (**A**) Viability assessment using ViaDye Red. (**B**) Quantification of mitochondrial mass (MTDR MFI) using MitoTracker Deep Red dye. (**C**) Measurement of mitochondrial reactive oxygen species (ROS) using MitoSOX Red dye. All data are expressed as the mean ± s.e.m. Dots represent independent experiments. *P* values were calculated using one-way ANOVA with Dunnet’s *post-hoc* test.


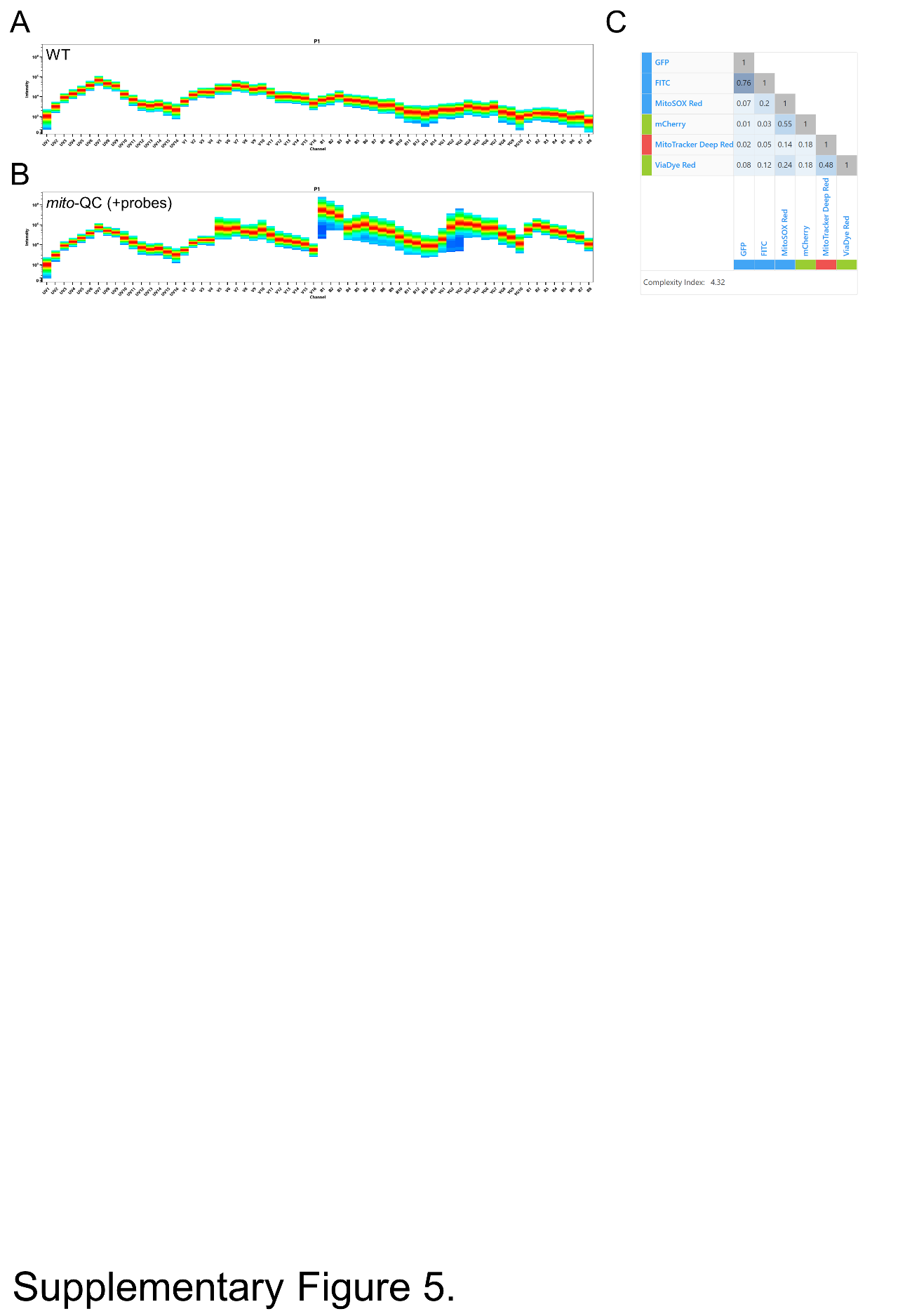


**Supplementary figure 5. Fluorescence spectra of wild-type and *mito*-QC ARPE-19 cells.** (**A**) Autofluorescence spectrum of wild type ARPE-19 cells in basal conditions (**B**) Fluorescence spectrum of *mito*-QC ARPE-19 cells multiplexed with intracellular probes (**C**) Similarity matrix of the fluorescence spectra of each fluorochrome and complexity index, which indicates the difficulty to resolve the panel.

| **Antigen specificity** | **Host species** | **Dilution** | **Supplier** | **Reference** |
| --- | --- | --- | --- | --- |
| BNIP3 | Mouse | 1:1000 | Abcam | ab10433 |
| BNIP3L/NIX | Rabbit | 1:1000 | Cell Signaling | 12396 |
| HIF1A | Rabbit | 1:1000 | Novus | NB100-479 |
| MT-CO1 | Mouse | 1:1000 | Invitrogen | 459600 |
| NDP52 | Rabbit | 1:1000 | GeneTex | GTX115378 |
| OPTN | Rabbit | 1:1000 | Abcam | ab23666 |
| Parkin | Rabbit | 1:1000 | Abcam | ab15954 |
| phospho-Ubiquitin(Ser65) | Rabbit | 1:1000 | Merck | ABS1513-I |
| PINK1 | Rabbit | 1:1000 | Cell Signaling | 6946 |
| SQSTM1/p62 | Mouse | 1:1000 | Abcam | ab56416 |
| TOMM20 | Rabbit | 1:2000 | Santa Cruz | sc-17764 |
| TOMM40 | Mouse | 1:1000 | Santa Cruz | sc-365467 |
| Vinculin | Rabbit | 1:1000 | Abcam | ab129002 |
| HRP Goat anti-Mouse | - | 1:4000 | Invitrogen | 31430 |
| HRP Goat anti-Rabbit | - | 1:4000 | Invitrogen | 31460 |

**Supplementary table 1. Antibodies used for immunoblotting.** Supplier and working dilution are indicated.
